# Research on optimization method of railway construction scheme based on multidimensional combination weighting and improved grey theory

**DOI:** 10.1038/s41598-023-50098-0

**Published:** 2024-02-06

**Authors:** Feng Han, Zelong Liu, Chengxiang Wang, Hao Wei, Bolin Wang

**Affiliations:** School of Civil Engineering, Lanzhou Jiaotong University, Lanzhou, 730070 China

**Keywords:** Engineering, Mathematics and computing

## Abstract

The optimization of railway construction schemes is a complexity system engineering task with multiple dimensions, diverse conditional constraints, and multifaceted objective assessments. The decision-making and scheme evaluation entail subjectivity, randomness, and fuzziness. To address the comprehensive optimization challenge in construction schemes effectively and efficiently, we investigate an optimization method for railway construction schemes. This method is based on multi-dimensional combination weighting and improved grey theory. After analyzing the primary influencing factors, we established a railway construction plan optimization index system comprising 4 dimensions and 18 factors. The weight combination coefficient is determined using the pros and cons solution distance method, and the optimal weight set for the index is determined through the multi-dimensional combination weighting approach. Utilizing the method of superior and inferior solution distance coupled with grey theory, we ascertain the order of advantages and disadvantages for each construction scheme, subsequently achieving construction scheme optimization. To illustrate this, we employ the optimization process for a high-speed railway section in Guangxi as an exemplar. The verification results indicate that the gray relative closeness values for schemes A, B, and C are 0.7089, 0.4813, and 0.4463, respectively. Scheme A has the highest gray relative closeness value, thus making it the optimal route scheme. The optimal results obtained through this method align with the outcomes of expert validation and existing research, thereby validating the effectiveness and practicality of the model. By employing a multidimensional combination weighting method, the deficiencies of traditional indicator weight calculations are mitigated, resulting in indicator weights that are more reflective of the actual circumstances. At the same time, the application of improvements in the grey theory comprehensive evaluation method enables the integration and computation of indicator data for each construction plan. Through the intuitive representation of grey relative closeness, the advantages and disadvantages of each plan are effectively characterized. This enhances the scientific rigor and applicability of the railway construction plan optimization process. The research findings can serve as a reference for similar railway construction scheme selection problems in the future.

## Introduction

The railway's construction has broken geographical limitations on economic flow. Despite the project's prolonged construction timeline and substantial investment, it assumes a pivotal role in enhancing the well-being of residents residing along the route and optimizing resource allocation. In light of a sequence of governmental policies introduced in recent years, it is evident that the national railway network will undergo further expansion and enhancement in the future. Consequently, numerous railway construction projects are confronted with the challenge of comparing and selecting among multiple construction schemes. The optimization of railway construction scheme is a complex system engineering with multiple objectives and multi-dimensional influencing factors^1^. Achieving scientific and rational optimization is a challenge under investigation by industry experts and scholars. In prior railway construction scheme selections, economic indicators were pivotal in assessing scheme merits and drawbacks. Engineering technology, social benefits, and environmental impact were typically translated into economic metrics. This leads to the fact that it is difficult to make an optimal judgment in the face of the corresponding indicators of each scheme and the small discrimination of economic indicators, and the optimization process is one-sided^2^.

The overarching analytical approach for railway construction scheme optimization is to establish an evaluation index system through a comprehensive analysis of influencing factors. Building on this foundation, the next steps involve selecting an appropriate calculation method to determine the weights for each index. Subsequently, you choose a suitable comprehensive evaluation method to process the data for each scheme. This process ultimately leads to the optimization of the scheme. In terms of index weighting, most of the initial use of a single weighting method, common expert evaluation method, Delphi method^3^, analytic hierarchy process^4^ and other subjective weighting method and entropy weight method^5^, coefficient of variation method^6^, CRITIC (Criteria Importance Tough Intercrieria Correlation) method^7^ and other objective weighting method. While the expert judgment method allows for the quick and convenient utilization of expert knowledge and experience, it is susceptible to the influence of individual experts' subjective biases. Additionally, it is constrained by the number of experts available and the scope of their domain knowledge. The Delphi method can reduce the subjective factors of experts, but it requires multiple rounds of surveys and a sufficient quantity of experts with high-quality participation. This leads to a longer timeframe and somewhat limited practical applicability. The Analytic Hierarchy Process (AHP) allows for the hierarchical analysis of complex problems. However, the subjectivity in the process of defining the hierarchy structure and pairwise comparisons is pronounced. Additionally, handling large-scale hierarchical structures can be quite complex and operationally challenging. The Entropy Weight Method can take into account the information and differences between indicators without relying on expert judgment. However, in cases where there is strong correlation between indicators, it may lead to weight bias. The coefficient of variation can account for the volatility and stability of indicators. However, in cases where there is low variability or similarity among the indicators, it may not be able to distinguish their weights effectively. The CRITIC method can comprehensively consider the relationships between indicators and their consistency with the decision objectives. However, in cases where the correlation between indicators is weak, it may not accurately reflect the weights. So, with the development of railway construction, a single weight is gradually difficult to meet the accuracy requirements, so the subjective–objective combination weighting method has emerged. To a certain extent, the combined weight reduces the subjective influence of human beings, and also reflects the law of objective data^8–10^. The combination weighting method has a great influence on the accuracy of the combination weight. The linear weighting method based on the distribution coefficient is the most commonly used, but there is still much room for improvement in the determination of the distribution coefficient. In terms of comprehensive evaluation methods, fuzzy comprehensive analysis method, set pair analysis method^11,12^, extension matter element analysis method^13,14^, TOPSIS (Technique for Order Preference by Similarity to an Ideal Solution) method^15^ and so on are used more. The fuzzy comprehensive analysis method, while capable of quantifying and handling fuzzy information, relies on fuzzy mathematical theory. It has complex algorithms and requires extensive computational resources. For large-scale complex problems, it may present challenges in terms of computation and model evaluation. The set pair analysis method transforms decision problems into a process of pairwise comparison and ranking, allowing for the quantification of the importance relationships between indicators. However, it has certain limitations when dealing with complex problems and situations where there are significant differences among multiple factors. The extension matter element analysis method comprehensively considers the relationships and uncertainties among evaluation indicators, making it capable of handling incomplete and uncertain information in decision problems. However, it may face certain challenges in data acquisition, requiring the collection and processing of extensive information. The TOPSIS method enables decision-making by comparing and ranking evaluation objects based on the weights of evaluation indicators. However, it cannot handle issues related to the correlation or mutual exclusivity among indicators. Additionally, it requires high standards for data standardization and normalization. Overall, these methods do not take into account the characteristics of independent, dynamic and uncertain railway construction indicators, so there are some limitations. In conclusion, it is necessary to employ scientifically sound new methods to establish models suitable for the optimal selection of railway construction schemes. The research presented in this paper is conducted based on this consideration.

Considering the current inaccuracy in weight determination and the insufficient depth of the comprehensive evaluation method. This paper employs an analysis of common weight calculation methods, including the hierarchical variable weight method, entropy weight method, grey correlation analysis method, and improved CRITIC method, for multi-dimensional calculations. By employing the idea of using the distance method for ranking pros and cons, a combination of weights is determined to strike a balance between the advantages and disadvantages of the aforementioned methods. This approach aims to reduce the negative impact of human factors as well as the uncertainties and correlations among the indicators, resulting in weights that closely approximate real-world scenarios. At the same time, the index characteristics are considered by the method of coupling the grey theory with the distance method, and the optimization of the scheme is realized on the basis of the comparison of the grey relative closeness. This method allows for a comprehensive consideration of multiple indicators, thereby reducing the impact of information gaps and providing a comprehensive basis for decision-making. Additionally, the applicability of this method is broader compared to previous approaches. The optimal decision model is validated through its application to the construction scheme of a high-speed railway section in Guangxi.

## Construction of the evaluation index system

**Figure 1:**
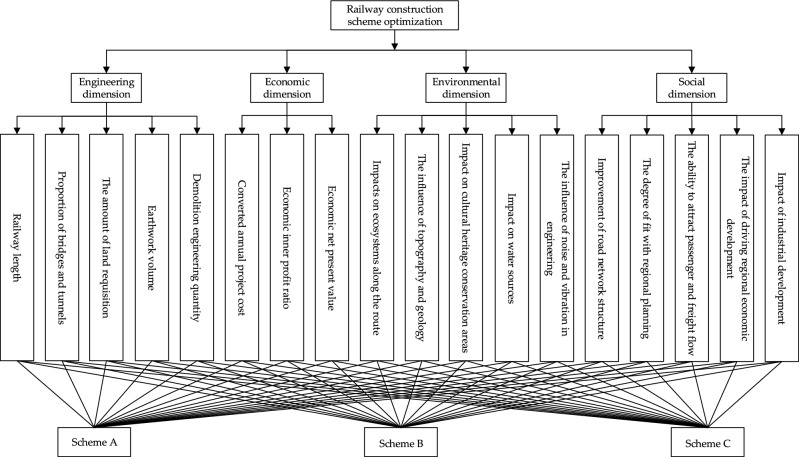
Optimize analytic hierarchy model diagram.

The evaluation and optimization of railway construction schemes represent a complex system engineering endeavor encompassing numerous contributing factors. A foundational requirement for achieving optimization is the establishment of a scientifically robust evaluation index system. Through literature research^15–17^ and expert consultation research methods, this paper starts from the engineering dimension, economic dimension, environmental dimension and social dimension, and combines the characteristics of railway construction and the information available in the early stage of railway construction. It is specifically refined into 18 aspects, such as railway length, proportion of bridge and tunnel, the amount of land requisition, earthwork volume, demolition engineering quantity, converted annual project cost, economic inner profit ratio, economic net present value, impacts on ecosystems along the route, the influence of topography and geology, impact on the cultural heritage conservation areas, impact on water sources, the influence of noise and vibration in engineering, the improvement of the road network structure, the degree of fit with regional planning, the ability to attract passenger and freight flow, the impact of driving regional economic development, and impact of industrial development. Then, the evaluation index system of railway construction scheme (Table 1) and the optimal analytic hierarchy model (Fig. 1) are constructed. The quantitative rules of qualitative indicators in the index system are shown in Table 2^18^.

**Table 1:** Evaluation index system of railway construction scheme.

Indicator number	Indicator	Indicator type	Indicator number	Indicator	Indicator type
1	Railway length	Quantification	10	The influence of topography and geology	Stationarity
2	Proportion of bridges and tunnels	Quantification	11	Impact on cultural heritage conservation areas	Stationarity
3	The amount of land requisition	Quantification	12	Impact on water sources	Stationarity
4	Earthwork volume	Quantification	13	The influence of noise and vibration in engineering	Stationarity
5	Demolition engineering quantity	Quantification	14	Improvement of road network structure	Stationarity
6	Converted annual project cost	Quantification	15	The degree of fit with regional planning	Stationarity
7	Economic inner profit ratio	Quantification	16	The ability to attract passenger and freight flow	Stationarity
8	Economic net present value	Quantification	17	The impact of driving regional economic development	Stationarity
9	Impacts on ecosystems along the route	Stationarity	18	Impact of industrial development	Stationarity

**Table 2:** Qualitative evaluation index quantification.

Interval number	[0, 1.0]	[1.0, 3.0]	[3.0, 4.5]	[4.5, 5.5]	[5.5, 7.0]	[7.0, 9.0]	[9.0, 10.0)
Cost indicators	Very large	Larger	Large	Average	Small	Smaller	Very small
Benefit indicators	Very good	Better	Good	Average	Bad	Worst	Very bad

## Multi-dimensional combination weighting based on TOPSIS

### Hierarchical variable weight theory

The analytic hierarchy process theory only considers the relative importance of various influencing factors in decision-making, but ignores the preference of state equilibrium degree. The variable weight theory is essentially a dynamic modeling principle. Considering that the index weight should change with the change of the index value state, in order to eliminate the deviation effect of the constant weight on the actual decision-making in the case of extreme state values^19^.

The expert judgment matrix $A = (z_{ij} )_{a \times a}$ obtained by the analytic hierarchy process^20^, and then the judgment matrix is processed by Eqs. (1) and (2) to obtain the constant weight of each index.

1 $$ w_{{_{i} }}^{0} = \frac{{\sqrt[a]{{\prod_{j = 1}^{a} z_{ij} }}}}{{\sum\limits_{i = 1}^{a} {\sqrt[a]{{\prod_{j = 1}^{a} z_{ij} }}} }} $$

2 $$ \left\{ \begin{gathered} \lambda_{\max } = \sum\limits_{i = 1}^{a} {\frac{{(AW)_{i} }}{{aW_{i} }}} \hfill \\ CR = \left( {(\lambda_{\max } - a)/(a - 1)} \right)/RI \hfill \\ \end{gathered} \right. $$

Combining the existing variable weight theory research and the research content of this paper^21,22^, the variable weight formula suitable for railway construction scheme optimization is obtained as shown in Eq. (3).

3 $$ w_{i} \left( {x_{1} ,x_{2} , \ldots ,x_{{\text{a}}} } \right) = \frac{{w_{i}^{0} x_{i}^{\alpha - 1} }}{{\sum\limits_{k = 1}^{{\text{a}}} {w_{k}^{0} x_{k}^{\alpha - 1} } }} $$

In the equation,$w_{i}^{0}$, $x_{i}$, $a$ and $w_{i}$ are the constant weight, state value, number of indexes and variable weight of each index respectively. α is the equilibrium coefficient, and α is generally 0.2–0.3 in engineering.

### Entropy weight method

The entropy weight method calculates index weights based on entropy values. Weight determination relies on the dataset, mitigating subjective influence in the calculation process and accurately reflecting data characteristics. The specific calculation is shown in formulas (4)–(6)^23^.

According to the line engineering information, the judgment matrix $E = ({\text{e}}_{ij} )_{a \times b}$ is constructed, and the standardized matrix $S = (s_{ij} )_{a \times b}$ is obtained by using the formula (4).

4 $$ s_{ij} = e_{ij} /\sum\limits_{i = 1}^{a} {e_{ij} } $$

The information entropy ${\text{e}}_{j}$ of each index is calculated by Eq. (5), and the entropy weight $w$ of the index is calculated by Eq. (6).

5 $$ e_{j} = - \frac{1}{\ln a}\sum\limits_{i = 1}^{a} {s_{ij} \ln } s_{ij} $$

6 $$ w = \left( {1 - e_{j} } \right)/\sum\limits_{i = 1}^{b} {(1 - e_{j} )} $$

### Grey relational analysis

Grey correlation analysis can determine the degree of similarity or dissimilarity between the two sets of system vectors^24^. According to the grey correlation analysis, the correlation coefficient between each index score vector and the relatively optimal index score vector can be calculated. Then the correlation degree of each index is calculated, and finally the correlation degree is normalized as the weight of each index.

The judgment matrix $E = ({\text{e}}_{ij} )_{a \times b}$ is sorted out to determine the reference data column $X_{0}$.

7 $$ X_{0} = (X_{0} (1),X_{0} (2), \ldots ,X_{0} (b)) $$

The absolute difference $C_{ij}$ between the evaluated object sequence and the reference sequence is calculated, and the maximum value is determined by using formulas (8) and (9).

8 $$ C_{ij} (\min ) = \mathop {\min }\limits_{i = 1}^{a} \mathop {\min }\limits_{k = 1}^{b} \left| {X_{0} (k) - X_{i} (k)} \right| $$

9 $$ C_{ij} (\max ) = \mathop {\max }\limits_{i = 1}^{a} \mathop {\max }\limits_{k = 1}^{b} \left| {X_{0} (k) - X_{i} (k)} \right| $$

Using formula (10), the correlation coefficients of the corresponding values of each comparison object sequence and the reference sequence are calculated respectively.

10 $$ \zeta_{i} (k) = \frac{{\mathop {\min }\limits_{i = 1}^{a} \mathop {\min }\limits_{k = 1}^{b} \left| {X_{0} (k) - X_{i} (k)} \right| + \rho \mathop {\max }\limits_{i = 1}^{a} \mathop {\max }\limits_{k = 1}^{b} \left| {X_{0} (k) - X_{i} (k)} \right|}}{{\left| {X_{0} (k) - X_{i} (k)} \right| + \rho \mathop {\max }\limits_{i = 1}^{a} \mathop {\max }\limits_{k = 1}^{b} \left| {X_{0} (k) - X_{i} (k)} \right|}} $$

In the formula, $\rho$ is the resolution coefficient. If $\rho$ is larger, the difference between the correlation coefficients is smaller, and the discrimination ability is weaker. In general, $\rho$ takes 0.5^25^.

According to formula (11), the correlation degree of each index is calculated respectively, and the grey correlation analysis weight of each index in formula (12) is used.

11 $$ r_{i}^{0} = \frac{1}{b}\sum\limits_{k = 1}^{b} {\zeta_{i} } (k) $$

12 $$ w_{i} = r_{i}^{0} /\sum\limits_{i = 1}^{a} {r_{i}^{0} } $$

### Improved CRITIC weight method

The judgment matrix $E = ({\text{e}}_{ij} )_{a \times b}$ is first transposed, and then standardized to obtain a standardized matrix $H = (h_{ij} )_{b \times a}$, in which Formula (13) is used for cost indicators and Formula (14) is used for benefit indicators.

13 $$ h_{ij} = \left( {\max (e_{ij} ) - e_{ij} } \right)/\left( {\max (e_{ij} ) - \min (e_{ij} )} \right) $$

14 $$ h_{ij} = \left( {e_{ij} - \min (e_{ij} )} \right)/\left( {\max (e_{ij} ) - \min (e_{ij} )} \right) $$

The improved CRITIC method^26^ uses the coefficient of variation to measure the discrimination of the index information, and its calculation formula is shown in Formula (15).

15 $$ v_{j} = \left( {\sqrt {\frac{1}{b}\sum\limits_{i = 1}^{b} {(h_{ij} - \frac{1}{b}\sum\limits_{i = 1}^{b} {h_{ij} } )^{2} } } } \right)/\left( {\frac{1}{b}\sum\limits_{i = 1}^{b} {h_{ij} } } \right) = \frac{{\sigma_{j} }}{{\overline{h}_{j} }} $$

The conflict between the indexes is expressed by the conflict coefficient, and the calculation formula is shown in Formula (16).

16 $$ C_{j} = \sum\limits_{i = 1}^{b} {(1 - \rho_{ij} )} $$

In the formula, $j = 1,2, \ldots ,a$.

$\rho_{ij}$ is the correlation coefficient between the indicators, and its calculation formula is shown in Formula (17).

17 $$ \rho_{ij} = {\text{cov}} \left( {H_{i}^{\prime } ,H_{j}^{\prime } } \right)/\left( {\sigma_{i} ,\sigma_{j} } \right) $$

The amount of information $Q_{j}$ of each index is calculated, and according to the principle that the greater the amount of information, the greater the weight of the corresponding index, the corresponding weight is calculated by formula (18) and (19).

18 $$ Q_{j} = \sigma_{j} \sum\limits_{i = 1}^{b} {(1 - \rho_{ij} )} $$

19 $$ W_{j} = Q_{j} /\sum\limits_{j = 1}^{a} {Q_{j} } $$

### Combination weighting determines the optimal combination weight

In order to make the multi-dimensional weight distribution more reasonable, this study carried out a combination calculation based on hierarchical variable weight, entropy weight, grey correlation analysis weight and improved CRITIC method weight, and used TOSPIP method^27^ to combine the weights obtained by the four methods. TOPSIS method is to determine the optimal weight set $D^{ + }$ and the worst weight set $D^{ - }$ after the multi-dimensional weight is composed of the initial weight matrix $W$ of $r$ row and $s$ column. Calculate the distance $K_{i}^{{^{ + } }}$ and $K_{i}^{{^{ - } }}$ from the weight set of each method to the optimal weight set and the worst weight set. The relative progress $T_{i}$ of the weight set of each method to the ideal solution is determined. The combination coefficient $\eta_{i}$ of the weight set of each method is obtained by normalization, and the combination weight is obtained by Eq. (23). The application of the idea of the distance method of the superior and inferior solutions in the combination weighting avoids the lack of theoretical basis in the practical application of the traditional linear weighting method, which makes the calculation results closer to the actual situation and has strong operability.

20 $$ \left\{ {\begin{array}{*{20}c} {\left( {E_{j} \in B} \right)\left\{ {\begin{array}{*{20}c} {D_{{^{j} }}^{ + } = \max \left\{ {\left. {w_{ij} \left| {1 \le i \le r} \right.} \right\}} \right.} \\ {D_{{^{j} }}^{ - } = \min \left\{ {\left. {w_{ij} \left| {1 \le i \le r} \right.} \right\}} \right.} \\ \end{array} } \right.} \\ {\left( {E_{j} \in C} \right)\left\{ {\begin{array}{*{20}c} {D_{{^{j} }}^{ + } = \min \left\{ {\left. {w_{ij} \left| {1 \le i \le r} \right.} \right\}} \right.} \\ {D_{{^{j} }}^{ - } = \max \left\{ {\left. {w_{ij} \left| {1 \le i \le r} \right.} \right\}} \right.} \\ \end{array} } \right.} \\ \end{array} } \right. $$

21 $$ \left\{ {\begin{array}{*{20}c} {K_{i}^{{^{ + } }} = \sqrt {\sum\limits_{j = 1}^{s} {(w_{ij} - w_{{_{j} }}^{ + } )^{2} } } } \\ {K_{i}^{{^{ - } }} = \sqrt {\sum\limits_{j = 1}^{s} {(w_{ij} - w_{{_{j} }}^{ - } )^{2} } } } \\ \end{array} } \right. $$

22 $$ \left\{ \begin{gathered} T_{i} = K_{i}^{{^{ - } }} /\left( {K_{i}^{{^{ + } }} + K_{i}^{{^{ - } }} } \right) \hfill \\ \eta_{i} = T_{i} /\sum\limits_{i = 1}^{r} {T_{i} } \hfill \\ \end{gathered} \right. $$

23 $$ W_{c} = \eta_{1} W_{1} + \eta_{2} W_{2} + \eta_{3} W_{3} + \eta_{4} W_{4} $$

In the formula, $E_{j}$ is the jth evaluation index, where 1 ≤ j ≤ s; $B$ is the benefit index set, $C$ is the cost index set; $W_{c}$ represents the combined weight of each index. $W_{1}$, $W_{2}$, $W_{3}$ and $W_{4}$ are the hierarchical variable weight, entropy weight, grey correlation analysis weight and improved CRITIC weight of each index in turn.

## Establishment of railway construction scheme optimization evaluation model based on improved grey theory

### Fundamental principle

Grey theory draws data information from the system and makes full use of the internal relations between the information. Among them, grey relational analysis is the most commonly used. It is a quantitative method to analyze the correlation degree of each index factor in the grey system. It only judges the correlation degree of the development trend of the grey process according to the similarity degree of the geometric shape of the sequence curve. The TOPSIS method characterizes the advantages and disadvantages of the set to be evaluated by the distance between the set to be evaluated and the positive and negative ideal solution sets. It only considers the similarity of the location and does not take into account the dynamic characteristics of the evaluation index. Therefore, there are some limitations in the independent use of the two methods^28^. In order to reasonably and fully characterize the index characteristics and scientifically and comprehensively realize the scheme optimization, this paper uses the TOPSIS method coupled with the grey theory to establish the optimization evaluation model.

### Establishing the model of evaluating

According to the line engineering information, the initial evaluation matrix $E = ({\text{e}}_{ij} )_{a \times b}$ is determined. On this basis, the relative optimal scheme set $v_{0} = ({\text{e}}_{1}^{0} ,{\text{e}}_{2}^{0} , \cdots {\text{e}}_{b}^{0} )$ is determined according to the principle that the smaller the cost index is, the better the benefit index is. By using the formula (24) and combining with the relative optimal scheme set, the initial evaluation matrix is normalized, and the normalized matrix $E_{0} = ({\text{e}}_{{_{ij} }}^{s} )_{a \times b}$ is obtained. The grey correlation degree between the data set of each construction scheme and the corresponding index of the relative optimal scheme set can be calculated by Formula (25)^29^.

24 $$ {\text{e}}_{{_{ij} }}^{s} = {\text{e}}_{ij} /{\text{e}}_{{_{j} }}^{0} $$

25 $$ \gamma_{ij} = \frac{{\xi \mathop {\max }\limits_{i} \mathop {\max }\limits_{j} \left| {e_{ij} - 1} \right|}}{{\left| {e_{ij} - 1} \right| + \xi \mathop {\max }\limits_{i} \mathop {\max }\limits_{j} \left| {e_{ij} - 1} \right|}} $$

In the formula, $\xi$ is the resolution coefficient, $\xi \in (0,1)$, the value of this paper is 0.5.

The grey correlation degree matrix of the railway construction scheme optimization problem with $a$ candidate construction scheme and $b$ evaluation indexes is set up as formula (26).

26 $$ G = \left[ {\begin{array}{*{20}c} {\gamma_{11} } & {\gamma_{12} } & \cdots & {\gamma_{1b} } \\ {\gamma_{21} } & {\gamma_{22} } & \cdots & {\gamma_{2b} } \\ \vdots & \vdots & \ddots & \vdots \\ {\gamma_{a1} } & {\gamma_{a2} } & \cdots & {\gamma_{ab} } \\ \end{array} } \right]_{a \times b} $$

The weighted correlation matrix $G^{w}$ is obtained by combining the combined weight $W_{c}$ of the evaluation index with the correlation degree of each alternative and the relative optimal scheme. The positive and negative ideal solution sets are determined by using the superior and inferior solution distance method combined with matrix $G^{w}$, and the Euclidean distance from each scheme to the positive and negative ideal solution sets is calculated, and then the grey relative closeness degree of each scheme is calculated (the specific calculation formula is similar to section of research about combination weighting deter-mines the optimal combination weight, which is not repeated here). According to the physical meaning of the grey relative closeness degree, the greater the grey relative closeness degree of the alternative scheme, the closer the index of the scheme to the relatively optimal scheme. Therefore, when the scheme is selected, the alternative scheme corresponding to the maximum gray relative closeness is the optimal scheme.

27 $$ G^{w} = \left[ {\begin{array}{*{20}c} {w_{1} \gamma_{11} } & {w_{2} \gamma_{12} } & \cdots & {w_{b} \gamma_{1b} } \\ {w_{1} \gamma_{21} } & {w_{2} \gamma_{22} } & \cdots & {w_{b} \gamma_{2b} } \\ \vdots & \vdots & \ddots & \vdots \\ {w_{1} \gamma_{a1} } & {w_{2} \gamma_{a2} } & \cdots & {w_{b} \gamma_{ab} } \\ \end{array} } \right]_{a \times b} $$

## Engineering examples

### General situation of the scheme

**Figure 2:**
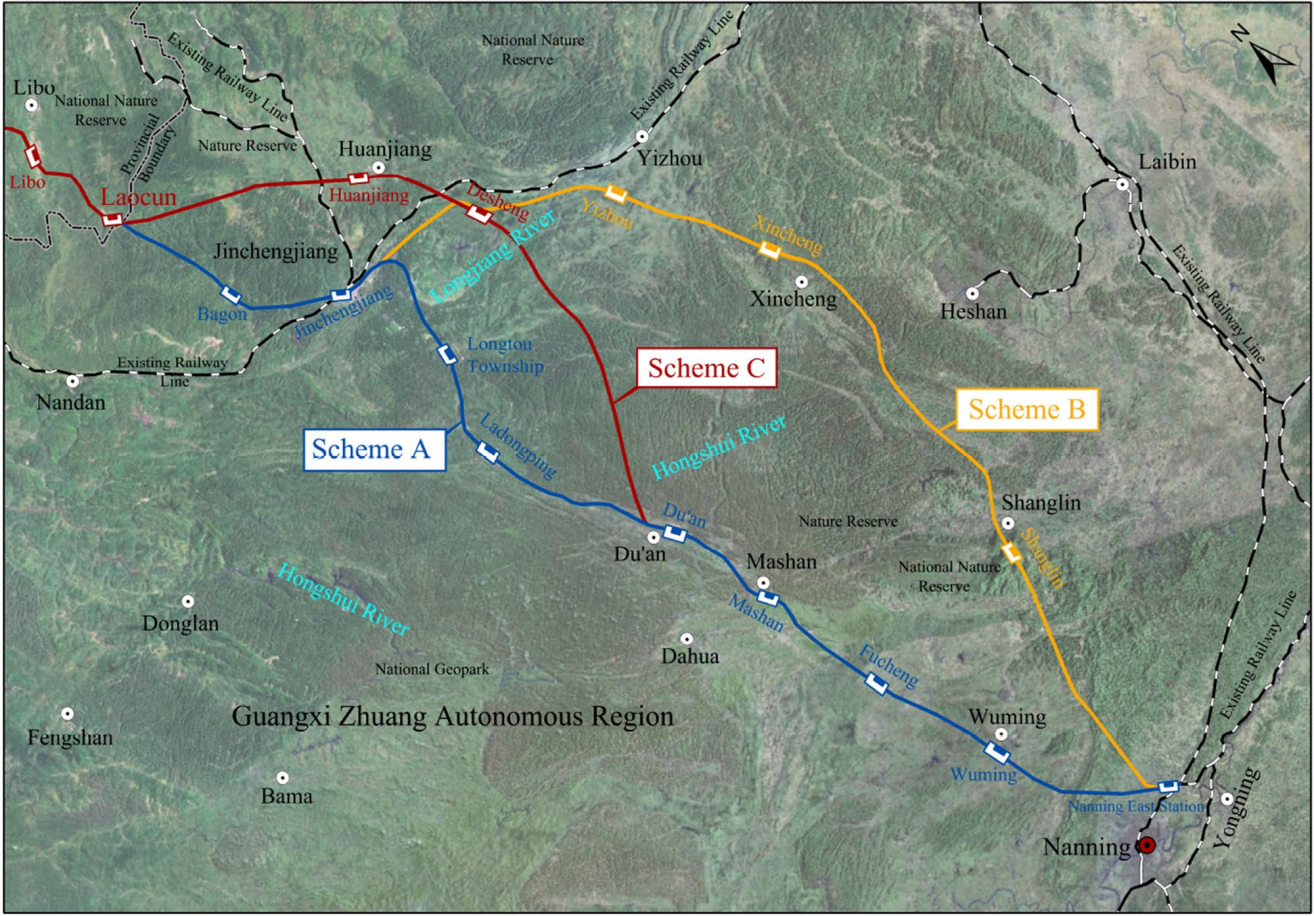
Illustration of construction scheme.

In order to better explain the optimization method of railway construction scheme based on multi-dimensional combination weighting improved grey theory, this paper selects the optimization example of a high-speed railway section construction scheme in Guangxi in reference^30^ to analyze the engineering application of the optimization model. The area where the railway passes through is mostly karst area, and the biodiversity along the railway is rich. At the same time, there are many environmental sensitive areas such as nature reserves, scenic spots, multi-type parks, water source protection areas, cultural relics and historical sites, and national key protected animals and plants along the railway. After the preliminary comprehensive comparison and analysis of this section of the railway, three alternative construction schemes are determined, which are Scheme A, Scheme B and Scheme C shown in Fig. 2. Regarding the generation and acquisition of Fig. 2, it is important to note that we obtained the foundational satellite imagery, including the study area, from publicly available datasets on the 'Geospatial Data Cloud' website. We performed the segmentation of the actual study area's satellite imagery using ArcGIS software. After segmentation, we incorporated the existing road network distribution within the study area, as well as the distribution of urban and environmentally sensitive areas along the route (all of which are part of publicly available data). Additionally, we created schematic representations of the alignment of each construction scheme based on actual route data. The relevant engineering information of the three railway construction schemes is shown in Table 3. The overview of each route construction scheme is as follows: Scheme A: After leaving Laocun Village Station, the route enters the existing Jinchengjiang Station after passing through Bagongshe Station, where it shares the platform with the existing station and then continues southward through Du'an, Mashan, and Wuming before connecting to Nanning East Station. The total length of the route is 304.52 km. Scheme B: After departing from Laocun Village Station, the route passes through Huanjiang and Yizhou West Station, then continues through Xincheng and Shanglin, avoiding the Daming Mountain National Nature Reserve, before connecting to Nanning East. The total length of the route is 314.21 km. Scheme C: After departing from Laocun Village Station, the route proceeds through Huanjiang and DeSheng and continues directly to Du'an, where the route aligns with the Du'an to Nanning section of Scheme A. The total length of the route is 311.32 km.

**Table 3:** Railway construction scheme setting.

Criterion layer	Indicator layer	Alternative scheme		
		A	B	C
Engineering dimension	Railway length (km)	304.52	314.21	311.32
Engineering dimension	Proportion of bridge and tunnel (%)	63.47	62.66	64.83
Engineering dimension	The amount of land requisition (hm^2^)	33,826.21	35,920.25	35,141.83
Engineering dimension	Earthwork volume (10^4^ m^3^)	126.83	134.68	131.76
Engineering dimension	Demolition engineering quantity (m^2^)	432,768	454,224	446,448
Economic dimension	Converted annual project cost (billion yuan)	362.3788	376.8	373.2
Economic dimension	Economic inner profit ratio (%)	9.8	8.8	9.2
Economic dimension	Economic net present value (million yuan)	1,103,386	972,086	1,023,026
Environment dimension	Impacts on ecosystems along the route	Smaller	Smaller	Smaller
Environment dimension	The influence of topography and geology	Larger	Large	Larger
Environment dimension	Impact on the cultural heritage conservation areas	Small	Small	Small
Environment dimension	Impact on water sources	Smaller	Small	Small
Environment dimension	The influence of noise and vibration in engineering	Large	Small	Large
Social dimension	The improvement of the road network structure	Good	Good	Good
Social dimension	The degree of fit with regional planning	Bad	Bad	Worst
Social dimension	The ability to attract passenger and freight flow	Better	Good	Bad
Social dimension	The impact of driving regional economic development	Better	Worst	Worst
Social dimension	Impact of industrial development	Better	Better	Better

### Multidimensional combination weighting

By using the four weight calculation methods mentioned in Sections “Hierarchical variable weight theory–Improved CRITIC weight method”, the weight calculation is carried out in turn, and then the multi-dimensional combination weight calculation is carried out according to the principle of Section “Combination weighting determines the optimal combination weight”, and the weight of each index is obtained as shown in Table 4. According to Table 4, it is evident that the three evaluation criteria with the highest weight percentages are the impact on regional economic development, the influence of terrain and geology along the route on the project, and the capacity to attract passenger and freight traffic. Conversely, the criterion with a relatively lower weight percentage is the amount of demolition work.

**Table 4:** Multidimensional combination weighting calculation table.

Indicator number	*W*_1_	*W*_2_	*W*_3_	*W*_4_	Combination weight
1	0.0132	0.0002	0.0567	0.0047	0.0128
2	0.0484	0.0002	0.0579	0.0066	0.0216
3	0.0072	0.0008	0.0555	0.0876	0.0319
4	0.0196	0.0008	0.0555	0.0876	0.0348
5	0.0059	0.0005	0.0560	0.0071	0.0118
6	0.1275	0.0003	0.0563	0.0058	0.0393
7	0.0363	0.0025	0.0639	0.0196	0.0238
8	0.0211	0.0034	0.0651	0.0231	0.0216
9	0.0593	0.0003	0.0565	0.0061	0.0238
10	0.1612	0.1789	0.0463	0.1251	0.1415
11	0.0256	0.0005	0.0559	0.0072	0.0163
12	0.0313	0.0206	0.0561	0.0525	0.0363
13	0.0120	0.0696	0.0516	0.0781	0.0558
14	0.1171	0.0005	0.0604	0.0073	0.0380
15	0.0677	0.0890	0.0530	0.0893	0.0787
16	0.0230	0.1067	0.0515	0.1049	0.0787
17	0.1842	0.5244	0.0410	0.2761	0.3120
18	0.0394	0.0008	0.0608	0.0113	0.0213

**Figure 3:**
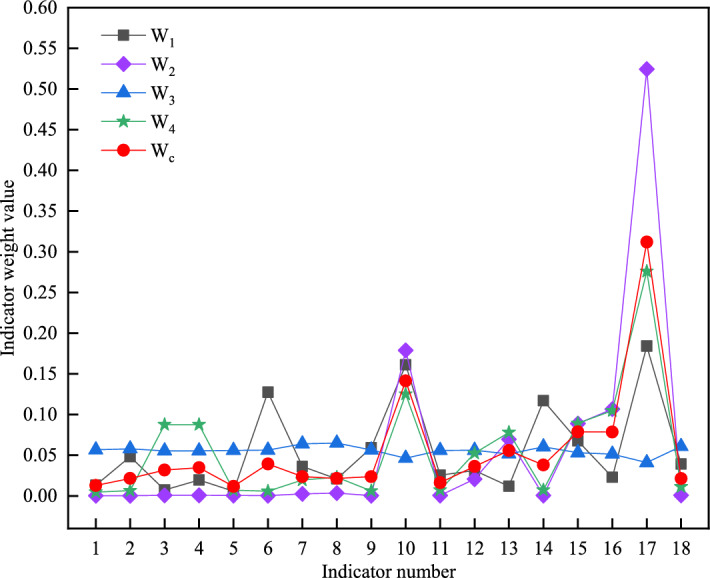
Comparison chart of evaluation index weight.

Figure 3 shows the results of index weight distribution under different weighting methods. It can be seen that the results of multi-dimensional combination weighting can avoid the extreme weight of some indexes in the weight distribution of each weighting method. It can also reasonably enlarge the degree of difference in information expression of evaluation indicators.

### Scheme evaluation and optimization based on improved grey theory

Combined with the quantitative rules of qualitative indicators in Table 2, the qualitative indicators in the setting of railway construction schemes are quantified, and the quantitative results are shown in Table 5.

**Table 5:** Quantitative results of qualitative indicators.

Indicator number	9	10	11	12	13	14	15	16	17	18
Indicator type	Cost indicators					Benefit indicators				
Scheme A	8.30	2.00	6.00	8.00	4.00	6.30	3.80	8.00	8.00	8.50
Scheme B	8.50	3.80	6.30	6.30	6.30	6.20	3.60	6.30	2.30	8.00
Scheme C	8.60	1.60	6.20	6.00	3.80	6.00	2.00	3.80	2.00	8.30

According to the quantitative description results obtained from the above table, combined with the known quantitative index data, the initial evaluation matrix is constructed as follows.

$$ \begin{gathered} E = \left[ {\begin{array}{*{20}c} {304.52} & {63.47} & {33826.2} & {126.83} & {432768} & {362.3788} & {0.098} & {1103386} & {8.30} \\ {314.21} & {62.66} & {35920.3} & {134.68} & {454224} & {376.8} & {0.088} & {972086} & {8.50} \\ {311.32} & {64.83} & {35141.8} & {131.76} & {446448} & {373.2} & {0.092} & {1023026} & {8.60} \\ \end{array} } \right. \hfill \\ \begin{array}{*{20}c} {} \\ {} \\ {} \\ \end{array} \begin{array}{*{20}c} {} & {} \\ {} & {} \\ {} & {} \\ \end{array} \left. {\begin{array}{*{20}c} {2.00} & {6.00} & {8.00} & {4.00} & {6.30} & {3.80} & {8.00} & {8.00} & {8.50} \\ {3.80} & {6.30} & {6.30} & {6.30} & {6.20} & {3.60} & {6.30} & {2.30} & {8.00} \\ {1.60} & {6.20} & {6.00} & {3.80} & {6.00} & {2.00} & {3.80} & {2.00} & {8.30} \\ \end{array} } \right] \hfill \\ \end{gathered} $$

According to the initial evaluation matrix, the relative optimal solution set $v_{0}$ is determined as follows.

$$ \begin{gathered} v_{0} = (\begin{array}{*{20}c} {304.52} & {62.66} & {33826.2} & {126.83} & {432768} & {376.8} & {0.098} & {1103386} \\ \end{array} \hfill \\ \begin{array}{*{20}c} {} & {} & {} \\ \end{array} \begin{array}{*{20}c} {8.30} & {1.60} & {6.20} & {6.00} & {3.80} & {6.30} & {3.80} & {8.00} & {8.00} & {8.50} \\ \end{array} ) \hfill \\ \end{gathered} $$

The formula (24) is used to standardize the initial evaluation matrix, and the standardized evaluation matrix is obtained as follows.

$$ \begin{aligned} E_{0} =  &   \left[ {\begin{array}{*{20}c} {1.0000} & {1.0130} & {1.0000} & {1.0000} & {1.0000} & {0.9617} & {1.0000} & {1.0000} & {1.0000} \\ {1.0318} & {1.0000} & {1.0619} & {1.0619} & {1.0496} & {1.0000} & {0.8980} & {0.8810} & {1.0241} \\ {1.0223} & {1.0347} & {1.0389} & {1.0389} & {1.0316} & {0.9904} & {0.9388} & {0.9272} & {1.0361} \\ \end{array} } \right. \hfill \\ \begin{array}{*{20}c} {} \\ {} \\ {} \\ \end{array} \begin{array}{*{20}c} {} & {} \\ {} & {} \\ {} & {} \\ \end{array}\\&\quad \left. {\begin{array}{*{20}c}   {1.0000} & {1.0130} & {1.0000} & {1.0000} & {1.0000} & {0.9617} & {1.0000} & {1.0000} & {1.0000} \\ {1.0318} & {1.0000} & {1.0619} & {1.0619} & {1.0496} & {1.0000} & {0.8980} & {0.8810} & {1.0241} \\ {1.0223} & {1.0347} & {1.0389} & {1.0389} & {1.0316} & {0.9904} & {0.9388} & {0.9272} & {1.0361} \\ \end{array} } \right] \hfill \\ \end{aligned} $$

The grey correlation matrix between each alternative and the relative optimal scheme is constructed by using Eq. (26), as follows.

$$ \begin{aligned} G & = \left[ {\begin{array}{*{20}c} {1.0000} & {0.9277} & {1.0000} & {1.0000} & {1.0000} & {0.8132} & {1.0000} & {1.0000} & {1.0000} \\ {0.9558} & {1.0000} & {0.9174} & {0.9174} & {0.9327} & {1.0000} & {0.8708} & {0.8525} & {0.9661} \\ {0.9438} & {0.9153} & {0.9060} & {0.9060} & {0.9223} & {0.9752} & {0.8596} & {0.8374} & {0.9121} \\ \end{array} } \right. \hfill \\&\quad \left. {\begin{array}{*{20}c} {} & {} \\ {} & {} \\ {} & {} \\ \end{array} \begin{array}{*{20}c}  {0.4000} & {0.8378} & {0.3333} & {0.7600} & {1.0000} & {1.0000} & {1.0000} & {1.0000} & {1.0000} \\ {0.3333} & {0.9771} & {0.9322} & {0.5110} & {0.9774} & {0.9289} & {0.7639} & {0.4911} & {0.9212} \\ {1.0000} & {1.0000} & {1.0000} & {1.0000} & {0.8873} & {0.4419} & {0.4167} & {0.3333} & {0.9410} \\ \end{array} } \right] \hfill \\ \end{aligned} $$

According to the weight and grey correlation matrix obtained by multi-dimensional combination weighting, the weighted correlation matrix between each scheme and the relative optimal scheme is calculated by Eq. (27). The results are shown in the following formula.

$$ \begin{aligned} G^{w} & =    \left[ {\begin{array}{*{20}c} {0.0128} & {0.0200} & {0.0319} & {0.0348} & {0.0118} & {0.0320} & {0.0238} & {0.0216} & {0.0238} \\ {0.0123} & {1.0216} & {0.0293} & {0.0319} & {0.0110} & {0.0393} & {0.0207} & {0.0185} & {0.0230} \\ {0.0121} & {0.0197} & {0.0289} & {0.0315} & {0.0108} & {0.0383} & {0.0204} & {0.0181} & {0.0217} \\ \end{array} } \right. \hfill \\ & \quad \begin{array}{*{20}c} {} \\ {} \\ {} \\ \end{array} \begin{array}{*{20}c} {} & {} \\ {} & {} \\ {} & {} \\ \end{array} \left. {\begin{array}{*{20}c}  {0.0566} & {0.0137} & {0.0121} & {0.0424} & {0.0380} & {0.0787} & {0.0787} & {0.3120} & {0.0213} \\ {0.0472} & {0.0159} & {0.0338} & {0.0285} & {0.0371} & {0.0731} & {0.0601} & {0.1532} & {0.0196} \\ {0.1415} & {0.0163} & {0.0363} & {0.0558} & {0.0337} & {0.0348} & {0.0328} & {0.1040} & {0.0201} \\ \end{array} } \right] \hfill \\ \end{aligned} $$

The TOPSIS is used to process the weighted correlation matrix, and the grey relative closeness of each scheme is calculated. The calculation results are shown in Table 6.

**Table 6:** Processing results of TOPSIS method.

Alternative scheme	Distance from positive ideal solution set	Distance from negative ideal solution set	Grey relative similarity degree	Recommended order
Scheme A	0.0896	0.2183	0.7089	1
Scheme B	0.1878	0.1743	0.4813	2
Scheme C	0.2176	0.1755	0.4463	3

The calculation results in Table 6 show that the grey relative closeness of scheme A is the largest, so scheme A is the optimal scheme. The advantages and disadvantages of scheme B and scheme C are similar, which are the second and third choices respectively. Therefore, according to the processing results, the construction scheme A is recommended.

### Verification analysis

Scheme A connects the most economic strongholds, the shortest railway length and the least investment. The station is located in the existing urban area, which is conducive to attracting passenger flow and rapid distribution, but the demolition project is complex and the long-term development is limited. Scheme B has many economic strongholds, which can promote the economic development of cities near the railway. However, the longest investment of the railway is the most, and the scope of attracting tourists overlaps with the nearby railways, which is not conducive to giving full play to the benefits of the railway. Scheme C passes through many economic strongholds, but the railway is far away from the existing urban area, which is not conducive to attracting passenger flow and rapid distribution. In summary, the expert demonstration result is recommended construction scheme A.

The expert’s actual demonstration of the railway construction plan and the judgment results in the literature^30^ are consistent with the judgment results of this study. This confirms the accuracy and effectiveness of the research on railway construction scheme optimization based on multi-dimensional combination weighting improved grey theory decision model proposed in this paper.

## Discussion

The evaluation and optimization of railway construction scheme is a complex decision-making problem involving multiple factors and multiple attributes. First of all, to establish a reasonable evaluation index system. But there is no uniform standard. At the same time, there is little engineering information in the early stage of railway construction, and the factors affecting route selection are closely related to cost, benefit, technology, safety and surrounding environment^31^. Due to the long construction period of the railway and the large number of crossing sensitive areas, unreasonable construction schemes will reduce its traffic efficiency and even lead to ecological and social problems. Once the environment along the railway is destroyed, later recovery is very difficult and expensive. Therefore, the construction of the evaluation index system of railway construction scheme should not only consider the railway construction itself, but also pay attention to the economic benefit factors, ecological environment factors and social stability problems. Hence, 18 indicators have been chosen, representing engineering, economic, environmental, and social dimensions, to form the foundation of the railway construction plan evaluation index system. It's crucial to acknowledge that the index system outlined in this paper has inherent constraints, primarily due to variations in the environment and relevant engineering conditions across construction areas. Therefore, when practically applied, adjustments to the construction scheme evaluation index system can be made to align with the specific characteristics of these regions.

At present, the general research idea is to determine the comprehensive weight of the evaluation index by combining the subjective and objective weight methods. The slight change of index weight will have a significant impact on the evaluation results of the scheme. Because there are some qualitative indicators in the evaluation index system, it is difficult to use the objective weighting method to evaluate the weight. At present, the weight methods commonly used in the comparison and selection of railway line schemes have their own advantages and disadvantages, and the overall consideration is not comprehensive enough. This study integrates and optimizes the four commonly used weight determination methods, and uses the TOPSIS method to reasonably combine the four weights to obtain a comprehensive weight that can not only reflect the objective information of the decision-making, but also reduce the impact of subjective factors on the decision-making results.

The optimization design of railway construction scheme is the key premise to reduce the impact of railway engineering on the original environment of the construction area. At present, the comprehensive evaluation of railway schemes is often divided into two parts: qualitative discussion of qualitative indicators by experts and quantitative calculation of quantitative indicators by designers. On the basis of the two, the comprehensive evaluation results are obtained. In fact, these methods do not achieve a good unified calculation of qualitative and quantitative indicators. The tedious calculation and complex model in traditional railway design greatly reduce the efficiency and accuracy of railway scheme optimization. Common methods in comprehensive evaluation include TOPSIS and Grey Theory. Both of these methods involve ranking decisions by calculating the similarity between indicator sequences. However, their areas of focus differ. The TOPSIS method focuses on the proximity of alternative solutions to the ideal solution, considering only positional similarity and not accounting for the dynamic changes in evaluation criteria. Grey theory primarily focuses on the correlation between alternative solutions and reference solutions, which can effectively reflect the internal patterns of change within each solution, but it only considers the similarity in terms of shape. Both of these methods have their respective advantages, but they also have limitations when used independently. Therefore, this study utilizes the TOPSIS method to improve and optimize grey theory in order to balance the limitations of both approaches, thereby achieving a more comprehensive and accurate comprehensive evaluation result. The improved grey theory realizes the optimization of railway construction scheme. Compared with the traditional optimization model theory, the model proposed in this study improves the efficiency and accuracy of calculation.

## Conclusions

This paper proposes an evaluation model for the optimization of railway construction schemes. The factors affecting railway design are analyzed from the engineering dimension, economic dimension, environmental dimension and social dimension, and the evaluation index system is constructed by selecting the main factors. The TOPSIS method is used to combine the four common weight calculation methods to obtain the comprehensive weight of the index. At the same time, the TOPSIS method is used to improve the grey theory, and the evaluation model of railway construction scheme optimization based on multidimensional and improved grey theory is established. Taking a railway section in Guangxi as an example, the validity of the model is verified. We can draw the following conclusions:

1. According to the actual situation of railway construction, this paper selects evaluation indexes from engineering dimension, economic dimension, environmental dimension and social dimension. Combined with the characteristics of relatively limited information in the early stage of railway construction, the four dimensions are expanded and refined to 18 evaluation indexes, and a simple and effective evaluation index system for railway construction scheme optimization is constructed.
2. The multi-dimensional combination weighting method is used to determine the optimal combination weight, which avoids the subjectivity in the process of weight determination. At the same time, the introduction of TOPSIS method ensures the scientificity and rationality of the combination coefficient. At the same time, the TOPSIS method coupled with the grey theory method is used to integrate the index data and analyze the advantages and disadvantages of the construction scheme, which avoids the ambiguity of human factors and traditional methods. The combination of multi-dimensional combination weighting and improved grey theory improves the accuracy and rationality of comprehensive evaluation.
3. Taking the optimization of the construction plan of a high-speed railway section in Guangxi as an example for verification analysis, the evaluation results are consistent with the expert demonstration results, which confirms the practicability and scientificity of the multi-dimensional combination weighting improved grey theory decision model. The model expresses the optimization process in a digital form, which has good practicality and applicability, and can provide some theoretical support for the optimization of subsequent railway construction schemes.

### Limitation of the study

The optimization of railway construction scheme is a systematic project involving many aspects. The scientific and reasonable construction of the evaluation index system is the key to the optimization of the scheme, but it is still in the exploratory stage. Therefore, the evaluation index system proposed in this paper has certain limitations, and the subsequent classification can be refined. In the future, we will conduct in-depth research on railway construction schemes in areas with clear attributes such as western mountainous areas, windy sand areas, and unfavorable geological areas.

## Data Availability

The data presented in this study are available on request form the corresponding author on reasonable request.
